# Decade-long analysis of postoperative endophthalmitis in Sweden: Insights from insurance and national quality registers

**DOI:** 10.1016/j.heliyon.2024.e38878

**Published:** 2024-10-04

**Authors:** Dalia Merkland, Andreas Viberg, Mattias Nilsson, Pelle Gustafson, Kristina Teär Fahnehjelm

**Affiliations:** a Division of Eye and Vision. Department of Clinical Neuroscience, Karolinska Institutet, Stockholm, Sweden; b Department of Retinal Diseases, St. Erik Eye Hospital, Stockholm, Sweden; c Department of Clinical Sciences/Ophthalmology, Umeå University, Umeå, Sweden; d The Swedish National Patient Insurance Company (Löf), Box 38069, Stockholm, Sweden; e Faculty of Medicine, Lund University, Lund, Sweden; f Department of Paediatric Ophthalmology, Strabismus and Electrophysiology, St. Erik Eye Hospital, Stockholm, Sweden

**Keywords:** Postoperative endophthalmitis, Ocular surgery, Infection, Insurance claims, Quality registry

## Abstract

**Purpose:**

To analyse clinical characteristics and risk factors for post-operative endophthalmitis (POE) in a Swedish patient insurance database and compare prevalence of POE with two national quality registers.

**Methods:**

We analysed 218 medical records of patients who claimed compensation for POE following ocular surgery from 2010 to 2020. The data were cross-referenced with national quality registers.

**Results:**

In total, 189 of 218 patients presented with severe ocular inflammation with no visibility into the posterior fundus and 160 of 218 had hypopyon. After treatment, the best corrected visual acuity improved from 2.4 to 1.8 LogMAR in mean (p < 0.01). Poorer visual outcome was associated with POE caused by *Staphylococcus aureus*, *Enterococcus faecalis*, and *Streptococcus* infections, as compared to coagulase-negative staphylococci (p < 0.01). Retinal detachment was linked to worse visual prognosis (p = 0.01). Eighteen patients required enucleation or evisceration, three of these had initially undergone vitrectomy, compared with 124 of 200 where the eyes were preserved. Peripheral vitrectomy showed better outcome compared to central vitrectomy (p = 0.02). Financial compensation was granted for 92 % of patients. However, a discrepancy was noted when comparing the number of patients with insurance claims to those reported by the national quality registers, indicating underreporting in both.

**Conclusion:**

This study highlights the impact of bacterial type on visual recovery in POE. Peripheral vitrectomy showed better outcomes than central vitrectomy. Most patients needing enucleation or evisceration were not treated with vitrectomy. Significant discrepancies were found between POE prevalence in quality registers and insurance reports, indicating the need for better reporting, though the exact magnitude of underreporting remains unclear.

## 1 Introduction

Postoperative endophthalmitis (POE), a severe intraocular infection, remains one of the most daunting complications following ocular surgery due to its potential for profound visual loss [[1], [2], [3], [4], [5]]. This underscores the importance of vigilant surveillance and improvements in clinical practices [6]. Generally, any surgery carries a risk of adverse events. However, most of these adverse events result from not applying best practices and are therefore considered avoidable. Patient safety aims to reduce the risk of avoidable adverse events, thereby optimizing outcomes in healthcare [7].

POE after ocular surgery is reported to two Swedish quality registers: the Swedish National Cataract Register (NCR) and the Swedish Macula Registry (SMR) [8,9]. The prevalence of POE according to the NCR after cataract surgery has been slightly above 0.02 % from 2008 to 2018 but declined in 2019 and 2020 to 0.01 % [9,10]. For intravitreal injections, the POE prevalence was 0.02 % in 2010 and 0.04 % in 2020 according to the SMR [11]. Both these registers, NCR and SMR, rely on healthcare-reported data. Another source, using patient-reported data, is the database of the Swedish National Patient Insurance Company (Löf), which provides patient insurance for all patients treated in health and dental care and covers over 95 % of all Swedish healthcare [12]. The Swedish Patient Injury Act ensures patients' rights to financial compensation for avoidable injuries [13].

This study aimed to analyse clinical characteristics and risk factors for POE including discrepancies in prevalence data using both the healthcare-reported data and medical records-based patient-reported data from Löf and from the two national quality registers.

## 2 Material and methods

We retrospectively analysed data from 218 patients who filed claims with Löf for endophthalmitis (ICD-10-SWE codes H44.0 and H44.1) during the period 2010 to 2020. Patients who had not undergone ocular surgery before the onset of endophthalmitis and those who declined participation in this study, either when filing a claim with Löf or declining registration in a quality register, were excluded (Fig. 2).

The research was conducted in adherence to the principles of the Declaration of Helsinki. Approval was obtained from the Ethical Review Board of Medicine (DR No. 2020–04583).

The NCR and SMR were searched for patients with reported POE. Swedish civil registration numbers in the Löf database were cross-referenced with those in the NCR and SMR to ensure comparability and data integrity.

All medical records and documentation in the claims, including patient demographics (age and gender), primary diagnosis, surgical procedure including prophylactic antibiotics, postoperative diagnosis, microbial pattern, clinical manifestations, antibiotic treatment for POE, and best corrected visual acuity (BCVA) outcome at up to 36 months follow-up were reviewed.

BCVA data was extracted from medical records and converted to the Logarithm of the Minimum Angle of Resolution (LogMAR). For patients with very low vision, the following conversions were applied: ‘counting fingers' = 1.85 LogMAR, ‘hand motion’ = 2.3 LogMAR, ‘light perception’ = 2.8 LogMAR, and ‘no light perception’ = 3.0 LogMAR [[14], [15], [16]].

Visual improvements were assessed using the Student's t-test and the Wilcoxon signed-rank test. The impact of the type of bacterial infection on visual outcome was analysed via ANOVA, while Pearson's correlation assessed the relationship between age and visual recovery. Complications and vitrectomy methods were compared using the independent *t*-test. Continuous variables were presented as means ± standard deviations (SDs), and categorical variables with frequency tables. Concordance between databases was evaluated using Cohen's kappa. All analyses deemed a p-value of <0.05 as statistically significant.

## 3 Results

### 3.1 Demographics

The study included 218 Löf-patients with a median age of 74 years (range 36–93). Of these, 113 patients (52 %) developed POE following cataract surgery, 86 (39 %) after intravitreal injection, 9 (4 %) after retinal surgery, 4 (2 %) after glaucoma surgery, and 6 (3 %) following other types of surgery. Among the 218 patients with POE, 89 (41 %) were aged 70–79 years. The gender distribution was 126 women (58 %) and 92 men (42 %).

Twenty-eight of the 218 surgical interventions (13 %) were associated with intraoperative complications, whereas in the other 190, no such complications were reported. The most common complication encountered during surgery was posterior capsular rupture, which occurred in 22 out of these 28 cases.

A weak negative correlation was seen between age at injury and changes in visual acuity, suggesting that as age at injury increases, the magnitude of visual acuity improvement decreases (Pearson correlation coefficient −0.15, p = 0.03). No gender-based differences were observed.

### 3.2 Clinical characteristics at presentation

Upon first clinical examination, 132 out of 218 patients (61 %) were reported to have exceedingly hazy media with no visibility of the posterior fundus. In these cases, B-scan ultrasonography was used prior to surgery to evaluate for retinal detachment, allowing visualization of the posterior segment despite the obscured view.

The ability to visualize the red reflex was observed in 57 out of 218 patients (26 %). Blurry insight into the ocular fundus was reported in 28 out of 218 patients (13 %), while only one patient was categorized as having good visibility into the retina on the first examination day.

The levels of inflammatory cells in the anterior chamber varied from very low to significant inflammatory responses. The most common observation was the presence of fibrin in 59 % of patients, indicating a substantial inflammatory response (Table 1). Hypopyon was present in 160 out of 218 patients (73 %), indicating significant inflammatory activity. In 56 patients, no hypopyon was found, and in two patients, information regarding hypopyon was missing.

### 3.3 Prophylactic treatment with topical antibiotics and POE treatment

During cataract surgery, as prophylactic treatment, 90 out of 113 patients (80 %) had been given intracameral cefuroxime, 15 patients (13 %) received moxifloxacin, and in six patients (5 %), antibiotic was given but the specific antibiotic used was not documented. There was no statistically significant association between type of prophylactic intracameral antibiotics and bacterial pattern.

POE treatment with intravitreal antibiotics was administered to 216 out of 218 patients (99 %). The most common antibiotic regimen was a combination of vancomycin and ceftazidime, used in 178 out of 218 patients, followed by a combination of vancomycin and gentamicin in 31 out of 218 patients. In one patient, the specific antibiotic used was not documented. Two patients did not receive antibiotic treatment due to poor ocular conditions upon arrival. (Table 2).

### 3.4 Visual and clinical outcomes

The median follow-up time was seven months (range 1–36 months). There was an improvement in BCVA following POE treatment. The mean LogMAR BCVA improved from 2.4 at arrival to 1.8 at long term follow up (p < 0.01).

Microbiological samples were obtained from the anterior chamber and vitreous for all patients. The most frequently identified bacteria were coagulase-negative staphylococci, found in 67 of 218 patients (31 %). Patients without bacterial growth showed the greatest mean improvement in BCVA (LogMAR), followed by those with coagulase-negative staphylococci infections. In contrast, infections caused by *Streptococcus*, *Enterococcus faecalis*, and *Staphylococcus aureus* were associated with poorer visual outcomes (*p* < 0.01). Across all bacterial groups, growth was more frequently detected in the vitreous. The ‘Other’ group contains a combination of these identified bacteria, along with isolated cases involving Serratia, Moraxella and Klebsiella (Table 3).

Retinal detachment after POE was linked to worse visual prognosis. The group without retinal detachment demonstrated a mean improvement of −0.67 LogMAR which was notably higher than the −0.095 LogMAR improvement observed in the group with postoperative retinal detachment (p = 0.01).

Regarding surgical interventions, among the 18 patients who underwent enucleation or evisceration due to POE, only three (17 %) did initially undergo vitrectomy. Conversely, among the remaining 200 patients who retained their eyes, 124 (62 %) underwent vitrectomy (p < 0.001).

**Fig. 1:**
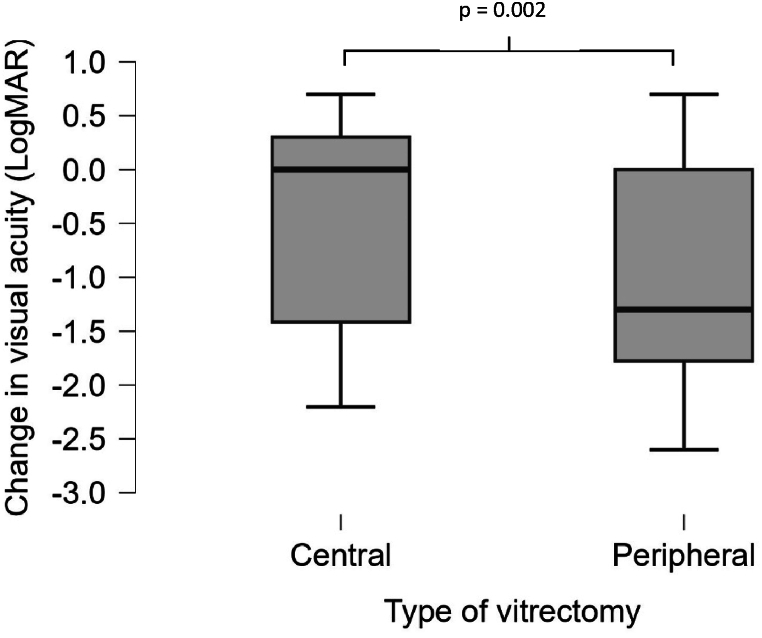
Boxplot: Differences in best corrected visual acuity (BCVA) in patients treated with peripheral vitrectomy vs central vitrectomy.

Analyses of differences in BCVA after surgical interventions comparing patients treated with peripheral vitrectomy to patients treated with central vitrectomy demonstrated significant differences. The group of patients with peripheral vitrectomy demonstrated a mean LogMAR improvement of −0.94, which was notably higher than the −0.36 improvement observed in the central vitrectomy group (p = 0.002) (Table 4). This suggests that peripheral vitrectomy was more effective in enhancing visual acuity among the patients studied (Fig. 1). There was no significant difference between the groups that underwent vitrectomy in terms of initial visual acuity or inflammation grade.

Financial compensation was approved for 201 out of 218 patients (92 %), based on the judgment that the condition was considered avoidable.

### 3.5 Discrepancies between different registers

**Fig. 2:**
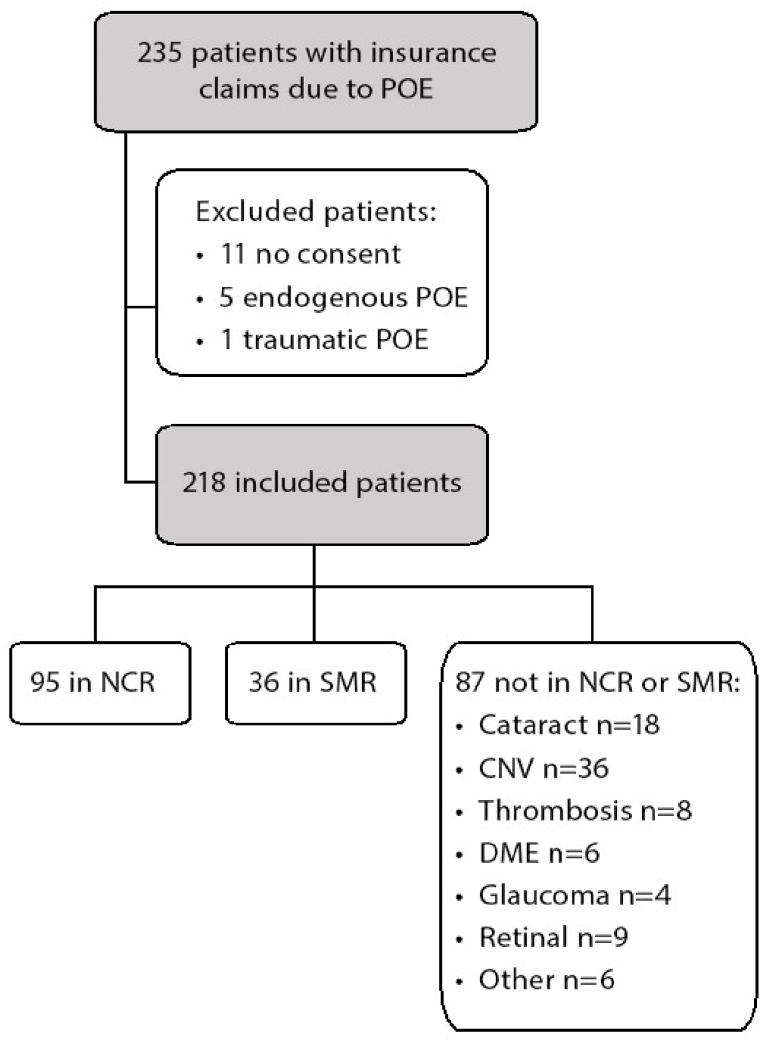
Illustrates the flow of patient selection and inclusion in the study. POE = Postoperative endophthalmitis. NCR = National Cataract Registry. SMR = Swedish Macula Registry. CNV = Choroidal neovascularization. DME = Diabetic macular edema.

**Fig. 3:**
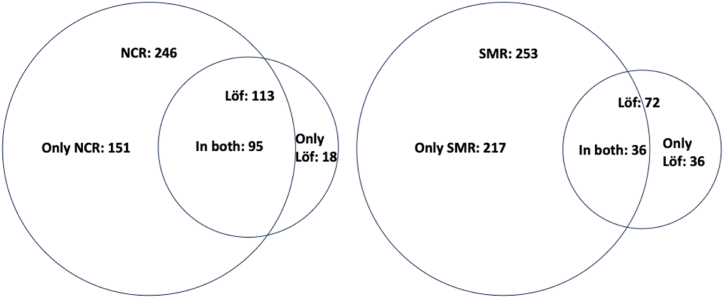
Proportional Venn diagram of correspondence between each quality register NCR (National Cataract Registry), SMR (Swedish Macula Registry) and Löf (Swedish National Patient Insurance Company).

A total of 218 patients from Löf were included in the present analysis Fig. 2, The NCR documented 246 patients with POE during the same period. Among these, 95 patients also submitted claims for economic compensation from Löf. Löf's database revealed an additional 18 patients with POE due to cataract surgery who had not been reported to the NCR. Thus, 95 out of 113 patients with POE in the Löf database due to cataract surgery were reported to the NCR (see Fig. 3).

The SMR reported 253 patients with POE during the study period. Among these, 36 patients also applied for compensation from Löf. Löf's database identified another 36 patients with POE related to the treatment of CNV who had not been reported to the SMR. SMR identified 36 out of 72 patients with POE in the Löf database.

The age distribution was similar across all three registries, with most patients experiencing POE falling within the age range of 70–79 years. However, the mean age was significantly lower among patients reporting to Löf compared with those reported to NCR and SMR. The mean age of the POE patients in the NCR was 76.9 years (SD 8.6) compared to 74.8 years (SD 9.1) in the Löf database (p < 0.05). The mean age in the SMR was 78.2 years (SD 9.1) compared to 74.6 years (SD 8.4) in the Löf database (p = 0.0019).

The gender distribution showed equal frequency of men with POE in the NCR was 54 % (n = 134) and 54 % (n = 61) in the Löf database. However, in the SMR, the frequency of men was 26 % (67 of 253) compared to 24 % (17 of 72) in the Löf database (p < 0.0001).

**Table 1:** Grading of inflammation in the anterior chamber upon first clinical examination in patients with post-operative endophthalmitis.

Grading of inflammatory cells and inflammation in the anterior chamber	Frequency (n)	Percent (%)
0.5+	1	0.5
1+	1	0.5
2+	8	4
3+	26	12
4+	53	24
Fibrin	128	59
Missing	1	0.5
Total	218	100

**Table 2:** Injection of intravitreal antibiotics after diagnosis of post-operative endophthalmitis.

Antibiotics given after diagnosis of endophthalmitis	Frequency (n)	Percent (%)
Vancomycin + Amikacin	4	2
Vancomycin + Cefacillin	1	0.5
Vancomycin + Ceftazidim	178	82
Vancomycin + Gentamicin	31	14
Cefuroxim + Ceftazidim	1	0.5
Missing	3	1
Total	218	100

**Table 3:** Bacterial growth in anterior chamber and/or vitreous and mean LogMAR difference in best corrected visual acuity after treatment with intravitreal antibiotic compared to arrival.

Bacterial Group	Frequency, n (%)	Mean LogMAR Difference	SD	Growth in Anterior Chamber, n (%)	Growth in Vitreous, n (%)	Growth in Both Anterior Chamber and Vitreous, n (%)	Not Precisely Documented Growth Location, n (%)
No bacterial growth	19 (9 %)	−1.2	0.957	N/A	N/A	N/A	N/A
Coagulase-negative staphylococci	67 (31 %)	−0.96	0.981	3 (5 %)	38 (57 %)	16 (24 %)	10 (15 %)
Streptococcus	29 (13 %)	−0.47	0.957	2 (7 %)	14 (48 %)	8 (28 %)	5 (17 %)
*Enterococcus faecalis*	53 (24 %)	−0.3	0.931	2 (4 %)	24 (45 %)	9 (17 %)	18 (34 %)
*Staphylococcus aureus*	24 (11 %)	−0.13	0.850	1 (4 %)	15 (62.5 %)	3 (12.5 %)	5 (21 %)
Other^a^	26 (12 %)	N/A	N/A	N/A	N/A	N/A	N/A

a The ‘Other’ group contains a combination of these identified bacteria, along with isolated cases involving Serratia, Moraxella and Klebsiella.

**Table 4:** Differences in best corrected visual acuity in patients with post-operative endophthalmitis treated with central vs peripheral vitrectomy.

Description	Group	N	Mean	SD	SE	Coefficient of Variation
Change in best corrected visual acuity LogMAR	Central	45	−0.36	0.987	0.147	−2.764
Change in best corrected visual acuity LogMAR	Peripheral	72	−0.94	0.982	0.116	−1.049

## 4 Discussion

In the current study, we reported the clinical characteristics of POE, its prevalence, and the reporting coverage by patients and healthcare personnel in the insurance database Löf compared with the two national quality registries, NCR and SMR.

More than 40 % of the Löf patients were between 70 and 79 years of age. A majority, 87 %, of the whole group of patients, presented with severe ocular inflammation, confirming the severity of the inflammatory response in POE cases. Demographically, increasing age at injury showed a weak correlation with poor visual acuity after POE, underscoring the potential impact of age-related physiological differences in healing and response to treatment. This finding is supported by other studies (Moshfeghiet al. 2004). However, our analysis did not reveal any gender-based differences in treatment outcomes [17].

Intraoperative complications were observed in 28 out of 218 surgical interventions that preceded POE (13 %). The most common complication encountered during surgery was posterior capsular rupture, which occurred in 22 out of these 28 cases. Posterior capsular rupture is a well-known complication in cataract surgery that can lead to vitreous loss, increased risk of retinal detachment, and potentially higher rates of postoperative infection, such as endophthalmitis.

This underscores the challenges and risks inherent to surgical procedures, even in controlled settings.

The high prevalence of fibrin and hypopyon (59 and 73 % respectively) as markers of severe inflammation and infection underscores the imperative need for clinicians to urgently incorporate comprehensive diagnostic and therapeutic regimens. Despite the severity of the intraocular infection, the long-term outcome after a mean of seven months was generally favourable.

During cataract surgery, prophylactic antibiotic administration was a common practice. Intracameral cefuroxime was the most frequently used antibiotic, administered to four of five patients. This preference is likely due to cefuroxime's established efficacy in reducing postoperative endophthalmitis. Moxifloxacin was given to 13 % of the patients, reflecting its role as an alternative prophylactic agent. For 5 % of the patients the specific antibiotic used were not documented, highlighting a need for record-keeping to ensure optimal patient care and facilitate future research.

The treatment of POE predominantly involved intravitreal antibiotics, administered to 99 % of the patients. The most commonly used regimen was a combination of vancomycin and ceftazidime, given to 91 % of the patients. This combination is favored for its broad-spectrum coverage against both Gram-positive and Gram-negative organisms. Another combination of vancomycin and gentamicin was used in 14 % of patients. Importantly, the antibiotics administered were found to be sensitive to the bacteria isolated from the samples, and patient records indicate that the antibiotics used were appropriate and effective in treating infections. However, two patients did not receive antibiotic treatment due to the severity of their ocular conditions, highlighting the critical need for timely intervention in severe POE cases.

There is a standard treatment protocol for POE, according to National Knowledge Guidelines from Swedish Ophthalmological Society typically involving prompt administration of intravitreal antibiotics and, in severe cases, vitrectomy. However, not all clinics have access to retinal surgeons or specialized equipment for performing vitrectomy. In such cases, patients are often referred to larger centers where vitrectomy can be performed. This variation in access to care may result in delays, potentially impacting outcomes, particularly in severe cases of endophthalmitis. The patients showed significant improvement in visual acuity after treatment, with a mean improvement of 0.6 LogMAR in BCVA. These findings highlight the efficacy of POE treatment in enhancing visual acuity in the long term. This improvement not only underscores the potential benefits of POE treatment but also provides a basis for its continued use and further investigation in clinical practice to enhance patient outcomes. Furthermore, our study indicated the superiority of peripheral over central vitrectomy in achieving better visual outcomes. This finding could significantly influence surgical decision-making, suggesting that peripheral vitrectomy should be considered preferentially to optimize visual results [1].

The study found that peripheral vitrectomy was associated with better visual outcomes compared to central vitrectomy (mean LogMAR improvement of −0.94 vs. −0.36; p = 0.002). However, the surgical approach was not always coherent or standardized across all cases. Instead, it was determined by the specific intraoperative conditions and the surgeon's ability to visualize the posterior segment. In cases where media opacities or significant inflammation obscured the fundus, central vitrectomy was sometimes the only feasible option.

Vitrectomy has previously shown several advantages in treating endophthalmitis. The Endophthalmitis Vitrectomy Study (EVS) found that immediate vitrectomy significantly benefitted patients with only light perception, tripling their chances of achieving 20/40 visual acuity. Diabetic patients with hand movement or better vision also showed improved outcomes compared to those undergoing vitreous tap and biopsy, likely due to the reduction of pathogens and inflammatory materials. Another study reported significant visual gains in patients with severe vision loss who underwent vitrectomy within 72 h of acute-onset infectious endophthalmitis, suggesting that it may help prevent complications like phthisis or panophthalmitis [18]. However, 8 % of the patients needed enucleation or evisceration of the infected eye. Notably, these patients rarely underwent vitrectomy during treatment, suggesting potential areas for a more active approach in performing vitrectomy.

Regarding the microbiological spectrum, the specific type of bacteria notably influenced visual outcome. Patients whose POE was caused by coagulase-negative staphylococci experienced the most substantial visual improvement after treatment, results aligning with previous studies [[19], [20], [21], [22]]. In contrast, infections due to *Staphylococcus aureus*, *Enterococcus faecalis*, and *Streptococcus* were associated with poorer visual outcomes, indicating challenges in treating endophthalmitis caused by these bacteria and highlighting the varied virulence among different pathogens [19]. This raises considerations regarding the potential utility of adding additional intraoperative antibiotics intracamerally during cataract surgery. On one hand, the addition of intraoperative antibiotics could provide means to target these serious infections more effectively [23]. On the other hand, it is important to recognize that such infections are generally rare occurrences and subjecting the majority of patients to additional intraoperative treatment may introduce unnecessary risks and contribute to the development of antibiotic resistance [24,25]. Additionally, prophylactic intravitreal injections of therapeutic agents have been explored as an option for managing infections, although this approach requires further [26]. Studies have shown limited efficacy of preoperative topical antibiotics in preventing this complication [27].

Complications such as retinal detachment were, not surprisingly, linked to worse visual prognosis in the current study which has been reported previously [28,29]. This emphasizes the importance of early treatment to minimize the risk of retinal detachment.

Most of the patients (92 %) in this Löf study did receive financial compensation. This reflects the recognition of endophthalmitis as a significant post-surgical complication and can be considered a compensable injury associated with the surgical procedure. The question arises if POE should automatically be considered an economically compensable injury, given that it can occur even when standard protocols are followed in detail, and no errors occur during the care process.

Underreporting to the insurance company Löf was seen when comparing to the national quality registries and vice versa. Löf's database revealed an additional 18 patients with POE due to cataract who were not reported by NCR, and 36 patients with POE related to the treatment of CNV who had not been reported to the SMR.

The discrepancies observed between the reporting of POE cases to quality registries and the submission of claims for economic compensation to Löf highlight potential underreporting issues. Although we identified that additional cases were recorded in the insurance database but not in the registries, and vice versa, our data were incomplete, which limits our ability to fully quantify the extent of this discrepancy.

Underreporting to Löf may reflect a lack of awareness among patients about their entitlement to compensation. The patients who claimed compensation and were approved were significantly younger than the patients in NCR and SMR. This is particularly concerning as economic support can play a crucial role in a patient's life, especially since POE can lead to significant visual impairment and thus reduced quality of life. Studies have shown that visual impairment significantly impacts various aspects of life, including functional abilities, economic stability, and social interactions, all contributing to a lower quality of life [30,31]. Enhancing both patient and health worker knowledge and awareness about Löf is a primary goal. Streamlining the compensation claim process to make it more accessible and less daunting for patients may increase the likelihood of affected individuals seeking the financial support they are entitled to. Informing patients that the Löf procedure does not accuse specific physicians, a common misinterpretation, may also reduce reluctance to make claims. Addressing the discrepancies between Löf and the national quality registers calls for increased reporting by health-care staff to the national registers. The responsibility to inform patients about the patient insurance also lies with health-care staff.

## 5 Strengths and limitations of the study

The strength of the current study is that we analysed data on clinical characteristics, microbiological patterns, antibiotics used, and various surgical interventions and techniques—all critical factors for determining visual outcomes for patients suffering from POE. The current study also underscores the critical role of microbial patterns in post-surgical best-corrected visual acuity and suggests specific surgical interventions that optimize patient outcomes after POE.

One possible limitation of the current study is the potential inaccuracies in the use of ICD codes in the medical records of patients who reported to Löf, which might have led to missed patient claims. Additionally, there is a risk that clinical data were divergent and incomplete, as the results were analysed retrospectively without a specific protocol followed by the clinicians. The precise number of POE cases could be more accurately determined by using data from NCR and SMR.

Although the current study has made significant contributions to understanding the management and clinical outcomes of POE, underscoring the critical role of microbial patterns for post-surgical best-corrected visual acuity, and suggesting specific surgical interventions to optimize patient outcomes after POE, further studies are needed. Future studies can explore the benefits of various surgical techniques, including the potential for intraoperative antibiotics, to establish best practices for preventing and managing endophthalmitis. Future research efforts should also focus on identifying high-risk patient populations who may benefit most from this approach while minimizing potential adverse consequences and the development of antibiotic resistance in the broader patient population. Furthermore, the prevention of infections through hygienic preventive routines to limit the presence of *Enterococcus faecalis* in the eye during and after surgery must also be examined.

## Statement of ethics and funding information

This study was conducted in accordance with the Declaration of Helsinki. Approval for the study was granted by the Ethical Review Board of medicine under DR No. 2020–04583.

Financial support was provided by Löf for conducting research in the patient safety field. The funding organization had no role in the design or conduct of this research. There are no other conflicts of interest.

## CRediT authorship contribution statement

**Dalia Merkland:** Writing – review & editing, Writing – original draft, Formal analysis, Conceptualization. **Andreas Viberg:** Writing – review & editing, Methodology, Formal analysis, Conceptualization. **Mattias Nilsson:** Methodology, Data curation. **Pelle Gustafson:** Writing – review & editing, Formal analysis, Data curation, Conceptualization. **Kristina Teär Fahnehjelm:** Writing – review & editing, Formal analysis, Conceptualization.

## Declaration of competing interest

All authors declare no conflict of interest, nor commercial associations regarding the content of this article.
